# Identification of Two Novel α_1_-AR Agonists Using a High-Throughput Screening Model

**DOI:** 10.3390/molecules190812699

**Published:** 2014-08-20

**Authors:** Fang Xu, Hong Chen, Xuelan He, Jingyi Xu, Bingbing Xu, Biyun Huang, Xue Liang, Mu Yuan

**Affiliations:** Research Center, Guangzhou Medical University, 195# Dongfengxi Road, Guangzhou 510182, China

**Keywords:** agonist, α_1_-ARs, HTS model, anti-proliferative activities, subtype-selectivity

## Abstract

α_1_-Adrenoceptors (ARs; 1A, 1B, and 1D) have been determined to perform different prominent functions in the physiological responses of the sympathetic nervous system. A high-throughput screening assay (HTS) was set up to detect α_1_-AR subtype-selective agonists by a dual-luciferase reporter assay in HEK293 cells. Using the HTS assay, two novel compounds, CHE3 and CHK3, were discovered as α_1_-ARs agonists in α_1_-ARs expressed in HEK293 cells. These compounds also showed moderate/weak anti-proliferative activities against tested cancer cell lines. The HTS assay proposed in this study represents a potential method for discovering more α_1_-AR subtype-selective ligands.

## 1. Introduction

α_1_-Adrenoceptors (ARs, α_1__A_, α_1__B_, and α_1__D_), a family of G–protein-coupled seven-transmembrane receptors, are involved in the regulation of the cardiovascular and central nervous systems [1]. Three native α_1_-AR subtypes (α_1__A_, α_1__B_, and α_1__D_), which have been previously cloned and pharmacologically characterized [2,3,4,5,6], participate in modulating a large number of physiological functions. These features make α_1_-ARs highly advantageous pharmacological targets for the treatment of numerous pathologies [7]. α_1_-ARs transmit extracellular signals by coupling with heterotrimeric Gs/q proteins, in which the α subunit mainly dissociates from the βγ dimeric subunit. The dissociated subunit can initiate a cascade of downstream secondary messenger pathways and eventually induce gene transcription by various response elements, including cAMP response element (CRE), activator protein-1 (AP-1), serum response element (NFAT-RE), serum response element (SRE), and nuclear factor-кB (NF-кB) [8,9].

Although *in vitro* binding studies and functional experiments have been used to assess compounds’ pharmacological profiles at α_1_-ARs, HTS-compatible methods for subtype-selective α_1_-ARs agonists/antagonists are still lacking [10,11]. Reporter gene systems have been used to detect the activation of specific signaling cascades, followed by monitoring of the reporter protein expression by its enzymatic activity, which is linked to a variety of colorimetric, fluorescent, or luminescent read-outs. Given its inherent sensitivity, large signal dynamics, and simple set up, the reporter assay platform has been widely used as a high-throughput homogenous assay for screening GPCR targets that are linked to cAMP or Ca^2+^ signaling [12]. Our group has continued to develop a high-throughput method for screening subtype-selective α_1_-ARs agonists/antagonists. In the present study, a high-throughput screening assay was set up, and two novel small-molecule α_1_-AR agonists were identified using this report assay system. The method passed stability and repeatability assessments, and its binding affinity was verified by classical agonists/antagonists (phenylephrine, naftopidil, prazosin, BMY7378, 5-methylurapidil, L-765314).

## 2. Results and Discussion

### 2.1. Expression of α_1_-ARs in HEK293 Cells

**Figure 1:**
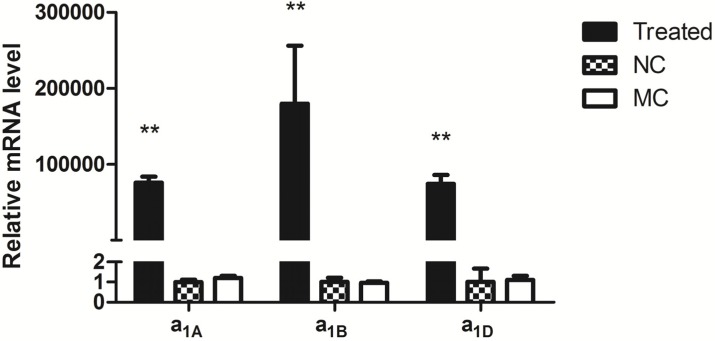
Expression of α_1_-ARs in transfected HEK 293 cells. Treated: transfected with α_1_-AR subtypes (α_1A_, α_1B_, and α_1D_) respectively; NC: negative control, HEK 293 cells only; MC: mock control, transfection reagent only. Data are represented as mean ± S.E.M. n = 3, ** *p* < 0.01.

The mRNA expression levels of three α_1_-AR subtypes were evaluated in HEK293 cells respectively by real-time RT-PCR at different time points. As shown in Figure 1, α_1_-ARs mRNAs were detectable at 24 h transfection. 

**Figure 2:**
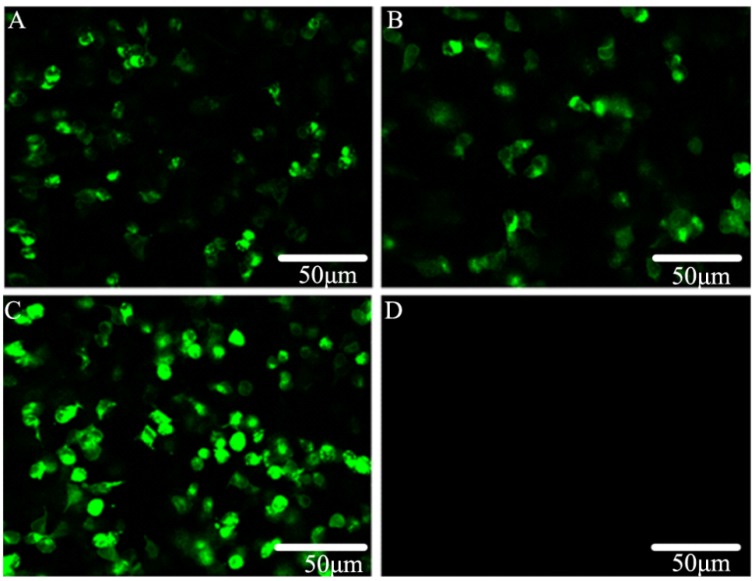
The transfected HEK 293 cells transiently transfected with α_1_-ARs 48 h respectively together with HEK293cells examined by inverted confocal microscope (Nikon, EZ-C1). (**A**) α_1A_-eGFP; (**B**) α_1B_-eGFP; (**C**) α_1D_-eGFP; (**D**) negative control, HEK 293 cells only.

It was shown that α_1_-ARs protein expressed with substantial fluorescence expression by transfected HEK293 cells (Figure 2A‒C). In contrast, there was no fluorescence expression by HEK293 cells (Figure 2D). These data suggest that α_1_-ARs gene successfully expressed in transfected HEK293 cells since 24 h transfection may mediate small-molecule-ligand endocytosis in the HTS system.

**Figure 3:**
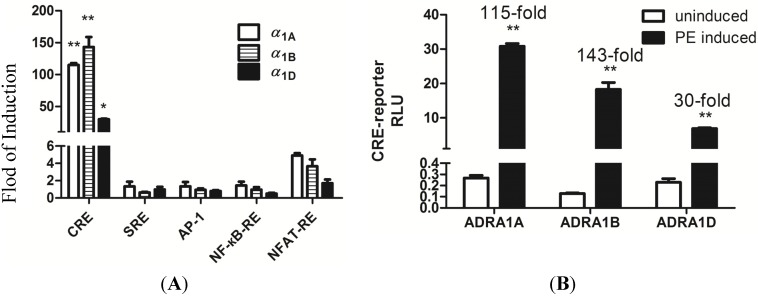
Transcriptional responses to different eukaryotic expression of human α_1_-ARs in HEK293 cells. (**A**) Transiently expressing various reporters (CRE, SRE, AP1, NF-кB, and NFAT) and α_1_-ARs (α_1A_, α_1B_, and α_1D_, respectively). (**B**) Transcriptional response to α_1_-ARs (α_1A_, α_1B_, and α_1D_, respectively) at the induction of PE (10 μM). Cells (uninduced) instead of 1% DMSO were used as control. Data are represented as mean ± S.E.M. n = 3, * *p* < 0.05, ** *p* < 0.01.

**Figure 4:**
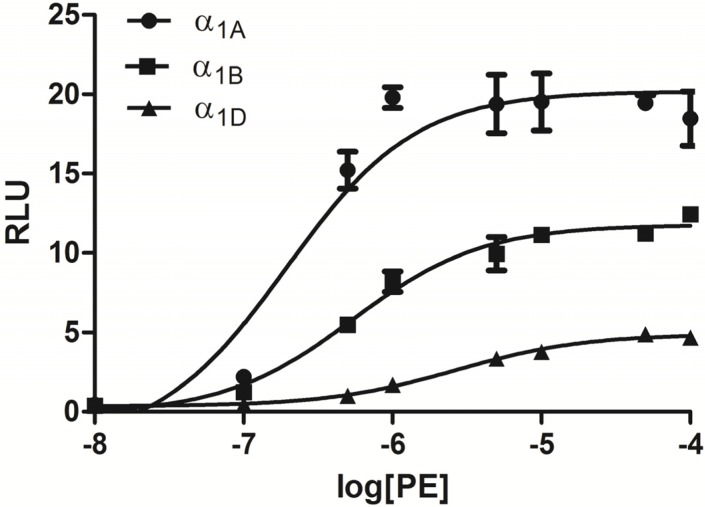
Transcriptional responses to PE in HEK293 cells transfected with α_1_-ARs (α_1A_, α_1B_, and α_1D_, respectively).

Characterization of dual-luciferase reporters with the eukaryotic expression of human α_1_-ARs (α_1A_, α_1B_, and α_1D_) was performed. To evaluate response elements, human ADRA1-expression vectors, namely, ADRA1A, ADRA1B, and ADRA1D, were co-transfected with CRE, SRE, AP-1, NFAT-RE, and NF-кB as response elements into HEK293 cells. These cells expressed α_1_-ARs at a high density and yielded different responses upon induction of phenylephrine (PE). As shown in Figure 3A CRE showed the highest activation with α _1_-AR (α_1A_, α_1B_, and α_1D_) participation in HEK293 cells, which are known to respond to Gs-coupled receptors [13]. Successful construction of the reporter expression vector was verified as pGL4.29 [luc2P/CRE] by screening for luciferase activity following treatment with an appropriate positive control (10 μM PE). To measure the activation of α_1_-ARs (α_1A_, α_1B_, and α_1D_) in PE (10 μM) in HEK293 cells, CRE was used to examine responses. Positive controls showed 115-, 143-, and 30-fold increases in luciferase activity in α_1A_, α_1B_, and α_1D_, respectively, in the CRE reporter gene relative to those in the blank control (Figure 3B). The effect of PE on each subtype of the transcriptional response reporter (CRE) expressed in HEK293 cells is shown in Figure 4. PE strongly activated the CRE reporter. The agonist PE has been demonstrated to induce dose-dependent expression of luciferase with distinguishable potencies. 

### 2.2. HTS Model Validation

**Figure 5:**
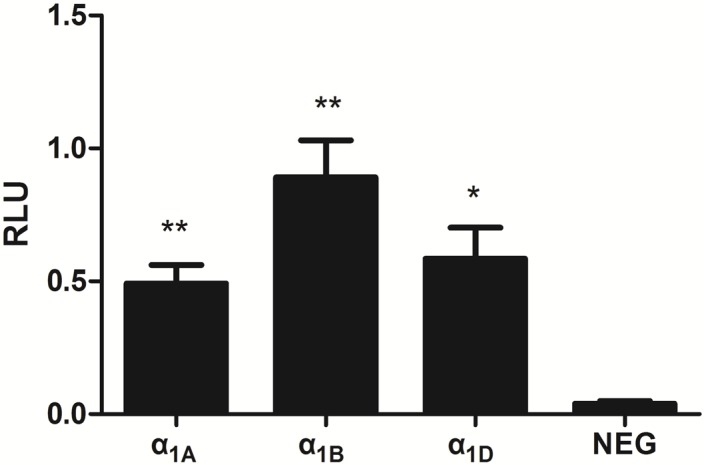
Transcriptional responses to PE in transfected HEK293 cells. Human ADRA1-expression vector: α_1_-ARs (α_1A_, α_1B_, and α_1D_) and negative control (NEG and EX-NEG-M29) respectively, co-transfected with CRE and pGL4.74 [hRluc/TK] (included as a control for transfection efficiency). Data are represented as mean ± S.E.M. n = 3, * *p* < 0.05, ** *p* < 0.01.

The negative control (NEG) vector was transfected by following the same procedure for α_1_-AR induction by PE. Figure 5 showed that CRE detects PE responses by α_1_-ARs rather than the opposite direction in cells. HEK293 cells expressing the luciferase reporter and receptors were induced in a 96-well plate with the agonist PE for 8 h in the response element CRE. Z'-factor values were then determined as 0.62, 0.68, and 0.73 by measurement of the relative luminescence units (RLUs) of the positive (PE + prazosin-induced) and negative (PE-induced) controls. The Z'-factor is typically used to evaluate assay performance during high throughput screening, and a Z'-factor value between 0.5 and 1 is acceptable for HTS; here, values closer to 1 indicate better assay quality [14].

### 2.3. HTS Assay for Agonists/Antagonists and Two Novel Agonists in Transfected HEK293 Cells: HTS Assay for Known Agonists/Antagonists of α_1_-ARs

**Figure 6:**
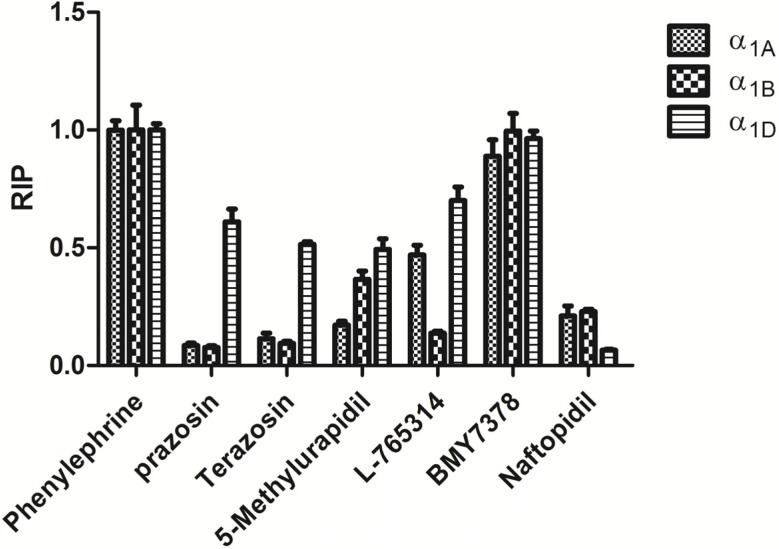
Luciferase activity assay data of known ligands on induced CRE activation in HEK 293 cells. Each point represented the mean ± S.D. of three individual experiments.

Six known α_1_-AR antagonists were validated using the HTS assay. Their inhibitory effects on PE-induced CRE production in α_1_-AR subtypes-HEK 293 cells are shown in Figure 6, where the relative light units (RLUs) were directly proportional to luciferase expression which indicated that RLUs could be used to evaluate the inhibitory efficiency of the compounds selectively inhibiting HEK 293-α_1_-ARs.

Furthermore, the relative inhibitory potency (RIP) could be used to improve the readability of the results. Which was determined relative to that of PE-induced group (negative control group), and calculated using the following formula [15]:

RIP = (RLU_PE alone_ − RLU_PE+antagonist_)/RLU_PE alone_ (1)

**Table 1:** Experimental RIP values with published subtype-selectivities of α_1_-ARs antagonists*.*

Compound	RIP Values (Mean ± S.E.M, n = 4)			Reported Selectivity
	α_1A_	α_1B_	α_1D_	
Prazosin	0.91 ± 0.02	0.92 ± 0.01	0.39 ± 0.11	Nonselective [16]
Terazosin	0.89 ± 0.05	0.91 ± 0.02	0.49 ± 0.02	Nonselective [17]
5-Methylurapidil	0.83 ± 0.03	0.63 ± 0.07	0.51 ± 0.09	α_1A_ > α_1D_ > α_1B_ [18]
L-765314	0.53 ± 0.08	0.86 ± 0.01	0.30 ± 0.11	α_1B_ > α_1D_ > α_1A_ [18]
Naftopidil	0.79 ± 0.09	0.77 ± 0.02	0.93 ± 0.01	α_1D_ > α_1A_ > α_1B_ [19]

The inhibited potency of the five compounds towards α_1_-ARs were estimated as in Table 1 [18,19,20]*.*

**Figure 7:**
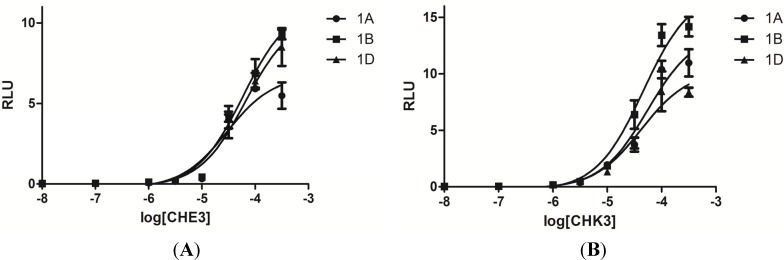
Agonistic activities with PE (10 μM) induced human α_1_-adrenoceptor subtype expressing HEK293 Cells. (**A**) CHE3; (**B**) CHK3.

The agonistic potency of CHE3 and CHK3 on α_1_-ARs were evaluated by the HTS assay and expressed as EC_50_ values (Figure 7, Table 2). As showed in Table 2, compounds CHE3 and CHK3 exhibited moderate agonistic activities (EC_50_ = 25.27–47.24 μM) on α_1_-ARs (α_1A_, α_1B_, and α_1D_). In addition, they did not display significant subtype selectivity in this assay.

### 2.4. In Vitro Cytotoxic Activity

Compounds CHE3 and CHK3 were evaluated for their *in vitro* cytotoxic activities against the three human prostate cancer cell lines (PC-3, LNCaP and DU145), and compared with their effects on human prostate epithelial cell line RWPE-1 by CCK-8 assay [19,20,21] using naftopidil as positive control [22]. As shown in Table 2, compounds CHE3 and CHK3 showed moderate/weak cytotoxic activities (IC_50_ = 11.07–91.27 μM) against tested cancer cell lines. However, these compounds exhibited cytotoxic effects on human epithelial prostate normal cells RWPE-1.

**Table 2:** Agonistic activities (EC_50_) on α_1_-adrenoceptor subtype (α_1A_, α_1B_, and α_1D_) and *in vitro* anti-proliferative activities (IC_50_) of CHE3 and CHK3.

Compound	EC_50_ (μM)			IC_50_ (μM) ^a^			
	α_1A_	α_1B_	α_1D_	PC-3 ^b^	LNCaP ^b^	DU145 ^b^	RWPE-1 ^b^
CHE3	25.27	42.67	47.24	91.27 ± 1.13	46.98 ± 1.24	11.07 ± 1.07	30.68 ± 0.66
CHK3	42.50	35.17	34.34	55.33 ± 1.09	51.50 ± 1.08	39.08 ± 0.81	31.67 ± 0.61
Phenylephrine ^c^	0.19	0.54	2.68	-	-	-	-
Naftopidil ^c^	-	-	-	42.10 ± 0.79	22.36 ± 0.61	34.58 ± 0.31	51.95 ± 1.51

^a^ IC_50_ values are taken as means ± standard deviation from three experiments. ^b^ PC-3, LNCaP and DU145, human prostate cancer cell line; RWPE-1, the human prostate epithelial cell line. ^c^ Used as a positive control.

## 3. Experimental Section

### 3.1. Materials

**Figure 8:**
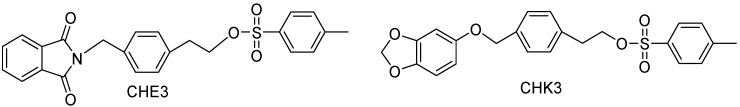
Chemical structures of CHE3 and CHK3.

HEK293 cells were purchased from Shanghai BIOLEAF Biotechnology Co., Ltd. (Shanghai, China). Reporter vectors: pGL4.29 [*luc2P*/CRE], pGL4.30 [*luc2P*/NFAT-RE], pGL4.32 [*luc2P*/NF-кB-RE], pGL4.33 [*luc2p*/SRE], pGL4.44 [*luc2P*/AP1 RE] and pGL4.74 [*hRluc*/TK] were obtained from Promega (Madison, WI, USA). Human ADRA1A/1B/1D-expression vector (NCBI: NM_000680.1/NM_000679.2/NM_000678.2) and negative control vector (NEG) were purchased from GeneCopoeia, Inc. Rockville, MD, USA; Catalog No.: EX-A0967-M29, EX-Y3321-M29, EX-Y2008-M29 and EX-NEG-M29). CHE3 and CHK3 [23,24] were supplied by the Pharmaceutical Research Center, Guangzhou Medical University. The chemical structures of CHE3 and CHK3 are shown in Figure 8. Other reagents were obtained from Sigma (St. Louis, MO, USA).

### 3.2. Cell Culture, Transfection and Administration

HEK293 cells were plated at a density of 10^5^ cells/cm^3^ in 96-well plates in 100 μL of DMEM basic medium containing 10% fetal bovine serum, 100 U/mL penicillin, and 0.1 mg/mL streptomycin. After overnight incubation, human ADRA1-expression vectors, namely, ADRA1A, ADRA1B, and ADRA1D, were co-transfected with reporter vectors (CRE, SRE, AP1, NFAT, and NF-кB) and pGL4.74 [hRluc/TK] (included as a control for transfection efficiency) using Lipofectamine 2000 transfection reagent (Invitrogen Life Technologies, Carlsbad, CA, USA). The medium was removed after 4 h and replaced with high-glucose DMEM for 18 h. Cells were then treated with agonists (at concentrations of 0.01–20 μM) and incubated for 8 h.

### 3.3. Dual-Luciferase Reporter Gene Assay

Firefly and Renilla luciferase activities, which are indicated as RLUs, were determined using Dual-Glo luciferase assay kits (Promega) according to the manufacturer’s instructions. RLUs were measured using a luminometer (GloMaxTM 96-Microplate Luminometer, Promega) and are reported as the mean ± SEM of three individual experiments. For agonists, fold of induction = RLUinduced/RLUuninduced. For antagonists, % of control = 100 × RLU (agonist + antagonist)/RLU(agonist alone). All RLUs were normalized against firefly RLUs/Renilla RLUs. Data are expressed as EC_50_/IC_50_ values in μM, and the ED50 of phenylephrine (μM) was calculated by plotting the data using nonlinear regression analysis in GraphPad Prism 5 software. The pKB of the antagonist was calculated by plotting log (dose ratio–1) against the antagonist dose (μM) and subjecting the data to Schild regression, where the intercept of the x-axis represents the pKB.

### 3.4. Fluorescence Microscopic Analysis

The expression of α_1_-ARs in HEK293 cells were detected by fluorescence microscopy. Human ADRA1-expression vector: ADRA1A, ADRA1B and ADRA1D were tagged by eGFP tag. The cells were treated as described above for α_1_-ARs HTS assay and observed using a confocal microscope (EZ-C1, Nikon, Tokyo, Japan)

### 3.5. Real-Time RT-PCR

HEK293 cells were grown in 6-well plates and transiently transfected with α_1_-ARs according to the protocol as described above. Total RNA was extracted and quantified followed the references using a spectrophotometer at 260/280 nm. Then cDNA was synthesized from total RNA by using oligo-dT primer. Each cDNA specimen and cloned human α_1_-AR subtypes SYBR Green PCR for each α_1_-AR subtype was performed by an Applied Biosystems 7500 Real Time PCR System. The primers and probes for α_1A_-, α_1B_-, and α_1D_-AR were designed as follows [25]: α_1A_-AR forward, 5'-ATCATCTCCATCGACCGCTACA-3'; a_1a_-AR reverse, 5'-TCACTTGCTCCGAGTCCGACTT-3'; a_1B_-AR forward, 5'-GCTCCTTCTACATCCCTCTGG-3'; a_1B_-AR reverse, 5'-AGGGTAGCCAGCACAAGATGA-3'; a_1D_-AR forward, 5'-ACCACGCGCAGCCTCGAGGCAGGC-3'; a_1D_-AR reverse, 5′-GAGCGAGCTGCGGAAGGTGTGGCC-3′. The PCR mix contained 2 μL cDNA template, 10 μL of 2× SYBR^®^*Premix Ex Taq* II (Takara, Shiga, Japan), 0.4 μL of 50× ROX Reference Dye II (Takara, 50×), 0.8 μL each of primers for α_1A_-, α_1B_-, or α_1D_-AR, in a total volume of 20 μL. GAPDH (glyceraldehyde-3-phosphate dehydrogenase) was used as internal control to standardize the quantitation of the three target genes. All experiments were carried out in triplicate.

### 3.6. Assessment of Antitumor Activity by CCK-8 Assay

Cell proliferation was measured with the Cell Counting Kit-8 (CCK-8) assay kit (Dojindo Corp., Kumamoto, Japan). Cells were harvested during logarithmic growth phase and seeded in 96-well plates at a density of 1 × 10^5^ cells/mL, and cultured at 37 °C in a humidified incubator (5% CO_2_) for 24 h, followed by exposure to various concentrations of compounds tested for 24 h. Subsequently 10 μL of CCK-8 (Dojindo) was added to each well, the cells were then incubated for an additional 1 h at 37 °C to convert WST-8 into formazan. Cell growth inhibition was determined by measuring the absorbance (Abs) at λ = 450 nm using amicroplate reader. Three independent experiments were performed. Cell growth inhibition was calculated according to the following equation:

Growth inhibition = (1 − OD of treated cells/OD of control cells) × 100%
(2)

The half maximal inhibitory concentrations (IC_50_) were obtained from liner regression analysis of the concentration-response curves plotted for each tested compound.

### 3.7. Statistical Analysis

Data are expressed as means ± standard errors. Differences among the mean values were analyzed by an unpaired Student’s *t* test. Values of *p* < 0.05 were considered to indicate statistical significance.

## 4. Conclusions 

The present study reported a rapid and sensitive assay for detecting α_1_-AR subtype-selective agonists/antagonists. Two novel compounds, CHE3 and CHK3, were discovered as α_1_-AR agonists using this assay. These compounds displayed moderate cytotoxic activities against tested cancer cell lines. The HTS assay proposed in this study represents a potential method for discovering more α_1_-AR subtype-selective ligands.

## Supplementary Materials

Supplementary materials can be accessed at: http://www.mdpi.com/1420-3049/19/8/12699/s1.

## Supplementary Files

Supplementary File 1
