# Supplementary material for: Identification of Two Novel α1-AR Agonists Using a High-Throughput Screening Model
Source: Molecules. 2014 Aug 20;19(8):12699–709. doi: 10.3390/molecules190812699 (PMC6270864; doi:10.3390/molecules190812699)
Supplement: Supplementary File 1 [file molecules-19-12699-s001.pdf]

Supplementary Materials

Figure S1. Luciferase activity assay data of phenylephrine (negative control), prazosin, terazosin, 5-methylurapidil, L-765314 and naftopidil on induced CRE activation in HEK293 cells. Each point represented the mean ± S.D. of three individual experiments.

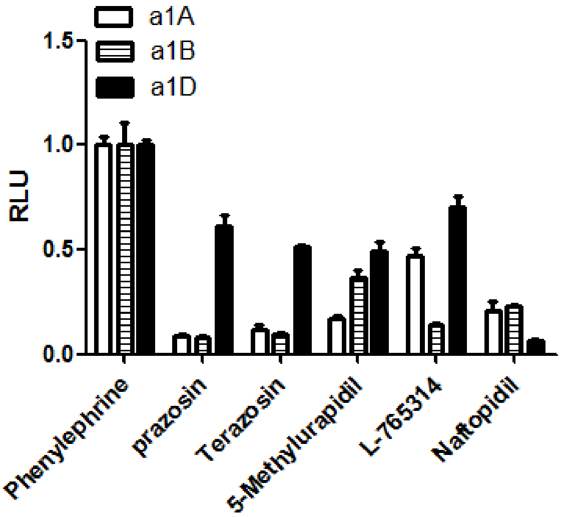

Table S1. Experimental RIP values with published subtype-selectivities of α_1-ARs antagonists.

**Compounds**	**RIP Values (Mean ± SEM, n = 4)**			**Reported Selectivity**
	α_{1A}	α_{1B}	α_{1D}	
Prazosin	0.91 ± 0.02	0.92 ± 0.01	0.39 ± 0.11	Nonselective [1]
Terazosin	0.89 ± 0.05	0.91 ± 0.02	0.49 ± 0.02	Nonselective [2]
5-Methylurapidil	0.83 ± 0.03	0.63 ± 0.07	0.51 ± 0.09	$\alpha_{1A} > \alpha_{1D} > \alpha_{1B}$ [3]
L-765314	0.53 ± 0.08	0.86 ± 0.01	0.30 ± 0.11	$\alpha_{1B} > \alpha_{1D} > \alpha_{1A}$ [3]
Naftopidil	0.79 ± 0.09	0.77 ± 0.02	0.93 ± 0.01	$\alpha_{1D} > \alpha_{1A} > \alpha_{1B}$ [4]

References

1. Sato, S.C.; Hatanaka, T.; Yuyama, H.; Ukai, M.; Noguchi, Y.; Ohtake, A. Taguchi, K.; Sasamata, M.; Miyata, K. Tamsulosin potently and selectively antagonizes human recombinant α1A/1D-adrenoceptors: Slow dissociation from the α1A-adrenoceptor may account for selectivity for α1A-adrenoceptor over α1B-adrenoceptor. *Biol. Pharm. Bull.* **2012**, *35*, 72–77.
2. Hillman, K.L.; Doze, V.A.; Porter, J.E. α_{1A}-adrenergic receptors are functionally expressed by a subpopulation of cornu ammonis 1 interneurons in rat hippocampus. *J. Pharmacol. Exp. Ther.* **2007**, *321*, 1062–1068.
3. Takei, R.; Ikegaki, I.; Shibata, K.; Tsujimoto, G.; Asano, T. Naftopidil, a novel α1-adrenoceptor antagonists, displays selective inhibition of canine prostatic pressure and high affinity binding to cloned human α1-adrenoceptors. *Jpn. J. Pharmacol.* **1999**, *79*, 447–454.
4. Yoo, T.K.; Cho, H.J. Benign prostatic hyperplasia: From bench to clinic. *Korean J. Urol.* **2012**, *53*, 139–148.
